# Supplementary material for: Eating Disorders in the Workplace
Source: Nutrients. 2025 Jul 12;17(14):2300. doi: 10.3390/nu17142300 (PMC12299150; doi:10.3390/nu17142300)
Supplement: Supplementary file 1 [file nutrients-17-02300-s001.zip › nutrients-3735621-supplementary.pdf]

Table S1. Collinearity statistics among variables used in the hierarchical multiple regression analysis reported in Table 3, Factors associated with suspected EDs. Tolerance and Variable Inflation Factor (VIF)

		Unstandardized Coefficients		Standardized Coefficients			Collinearity Statistics	
Model		B	Std. Error	Beta	t	Sig.	Tolerance	VIF
I	(Constant)	-.507	.710		-.715	.475		
	age	.028	.010	.063	2.762	.006	.981	1.019
	gender	1.262	.243	.118	5.194	.000	.983	1.017
	trauma	1.637	.305	.121	5.359	.000	.989	1.011
II	(Constant)	5.380	1.090		4.934	.000		
	age	.025	.010	.056	2.491	.013	.977	1.024
	gender	1.241	.241	.116	5.149	.000	.981	1.019
	trauma	1.617	.303	.120	5.327	.000	.988	1.012
	HLS	-.172	.025	-.185	-6.854	.000	.684	1.461
	DDL	.069	.048	.039	1.434	.152	.686	1.458
III	(Constant)	3.239	1.115		2.904	.004		
	age	.013	.010	.028	1.240	.215	.956	1.046
	gender	1.297	.239	.121	5.422	.000	.980	1.020
	trauma	1.344	.302	.100	4.453	.000	.976	1.025
	HLS	-.145	.025	-.155	-5.722	.000	.670	1.493
	DDL	.067	.048	.037	1.398	.162	.683	1.464
	ERI	2.161	.286	.173	7.551	.000	.938	1.066

IV	(Constant)	4.235	1.238		3.422	.001		
	age	.005	.010	.010	.472	.637	.945	1.059
	gender	.708	.235	.066	3.015	.003	.931	1.075
	trauma	.316	.300	.023	1.055	.292	.912	1.097
	HLS	-.086	.025	-.092	-3.500	.000	.648	1.543
	DDL	.072	.046	.040	1.554	.120	.682	1.466
	ERI	.326	.306	.026	1.063	.288	.746	1.340
	PSQI	.170	.048	.109	3.557	.000	.478	2.090
	anxiety	.201	.075	.102	2.666	.008	.305	3.281
	depression	.357	.088	.149	4.066	.000	.335	2.987
	happiness	-.174	.077	-.059	-2.260	.024	.647	1.545

a. Dependent Variable: EDs

Table S2. Collinearity statistics among variables used in the hierarchical multiple regression analysis reported in Table 3, Factors associated with suspected EDs. Eigenvalues and Condition index of each of the hierarchical models.

				Variance Proportions			
Model	Dimension	Eigenvalue	Condition Index	(Constant)	age	gender	trauma
I	1	3.819	1.000	.00	.00	.01	.01
	2	.086	6.659	.00	.05	.59	.34
	3	.075	7.137	.01	.41	.05	.54
	4	.020	13.878	.99	.54	.35	.12

Model	Dimension	Eigenvalue	Condition Index	(Constant)	age	gender	trauma	HLS	DDL
	1	5.731	1.000	.00	.00	.00	.00	.00	.00
	2	.091	7.949	.00	.00	.01	.74	.01	.10
	3	.085	8.217	.00	.11	.74	.00	.00	.03
	4	.065	9.356	.00	.54	.04	.17	.01	.16
	5	.021	16.616	.13	.21	.13	.04	.25	.64
II	6	.008	27.295	.87	.14	.09	.04	.73	.07

Model	Dimension	Eigenvalue	Condition Index	(Constant)	age	gender	trauma	HLS	DDL	ERI
	1	6.570	1.000	.00	.00	.00	.00	.00	.00	.00
	2	.168	6.261	.00	.00	.03	.00	.00	.02	.81
	3	.089	8.591	.00	.01	.03	.72	.01	.10	.02
	4	.083	8.897	.00	.07	.71	.13	.00	.02	.04
	5	.062	10.254	.00	.62	.02	.08	.00	.15	.07
	6	.021	17.876	.12	.19	.13	.04	.25	.64	.00
III	7	.007	29.937	.88	.10	.09	.03	.73	.07	.05

Model	Dimension	Eigenvalue	Condition Index	(Constant)	age	gender	trauma	HLS	DDL	ERI	PSQI	anxiety	depression	happiness
IV	1	9.381	1.000	,00	,00	,00	,00	,00	,00	,00	,00	,00	,00	,00
	2	.964	3.119	,00	,00	,00	,00	,00	,00	,00	,01	,04	,09	,00
	3	.145	8.051	,00	,00	,05	,01	,00	,00	,69	,00	,00	,14	,00
	4	.122	8.753	,00	,01	,00	,00	,00	,00	,07	,21	,35	,68	,00
	5	.103	9.527	,00	,02	,12	,04	,00	,00	,02	,55	,34	,01	,00

6	.085	10.522	,00	,00	,00	,85	,00	,02	,00	,10	,01	,02	,02
7	.072	11.391	,00	,02	,59	,00	,01	,12	,09	,08	,19	,00	,00
8	.062	12.343	,00	,56	,00	,02	,01	,19	,05	,04	,06	,01	,02
9	.044	14.600	,00	,22	,13	,01	,00	,15	,01	,00	,00	,04	,50
10	.016	24.591	,06	,06	,04	,04	,52	,50	,02	,01	,00	,01	,26
11	.006	38.691	,94	,10	,07	,04	,46	,03	,05	,01	,00	,01	,19
